# Supplementary material for: Influence of pin length on interfacial characteristics and tensile properties of friction-stir-butt-welded AA1060/Q235 clad plates
Source: PLoS One. 2026 Apr 8;21(4):e0346802. doi: 10.1371/journal.pone.0346802 (PMC13061233; doi:10.1371/journal.pone.0346802)
Supplement: Attachment — Submitted filename: Reviewer report (1).pdf [file pone.0346802.s001.pdf]

The paper presents an important study on the effect of tool pin length on the microstructure and mechanical properties of friction stir butt-welded AA1060/Q235 steel clad plates, yet several weaknesses limit its overall impact and scientific rigor. The abstract, while detailed, is overly descriptive and includes numerical data that belong in the results section. It also fails to emphasize the novelty or unique contribution of the study compared to existing works. Similarly, the introduction, though comprehensive, spends too much space explaining basic background information about Al/steel composites before identifying the research gap. References are occasionally dated or redundant, and the claim that research on Al/steel clad plate friction stir welding remains preliminary is not adequately supported with recent literature.

The literature review is embedded within the introduction, making it difficult to distinguish the authors' own reasoning from prior research. Although several relevant studies are cited, they are summarized mechanically without critical comparison or synthesis. The review also fails to articulate a clear knowledge gap, specifically it does not explain what remains uncertain about the role of pin length or how this study differs from prior works on similar FSW systems. Moreover, emerging developments such as hybrid, refill, or ultrasonic-assisted friction stir welding are not discussed, which would have provided a broader technological context for the research.

In the experimental section, the procedure is presented clearly but lacks justification for critical parameter choices such as rotational speed, traverse rate, and pin geometry. While the groove design and three-pass FSW strategy are described in detail, there is no explanation for how these choices were optimized or validated. Sample size, error margins, and replication are not discussed, which raises concerns about reproducibility. The section focuses extensively on surface preparation steps yet provides limited information on essential aspects like tool material, temperature control, or measurement accuracy. Moreover, there is no discussion of how the selected conditions might influence heat input, plastic flow, or IMC formation, all key variables in dissimilar metal welding.

The results and discussion section contain valuable observations supported by microscopy, EDS, and XRD analyses, but interpretation remains largely qualitative. Relationships between IMC thickness, pin depth, and tensile strength are mentioned but not quantified through statistical analysis or curve fitting. Figures are informative but inconsistently referenced and often lack scale bars or quantitative data. The mechanical analysis is superficial, with no detailed stress–strain characterization, finite element modelling, or error analysis to validate claims. Similarly, the explanation of fracture mechanisms relies solely on visual inspection of SEM morphologies without supporting quantitative evidence such as elongation or toughness data. The discussion does not integrate findings into a broader theoretical or industrial context, leaving readers uncertain about the general applicability of the results.

The conclusions restate observations rather than drawing clear engineering insights or guidelines for practice. There is no acknowledgment of limitations such as thermal gradients, measurement uncertainties, or tool wear, nor any recommendations for future work such as fatigue testing, temperature field modelling, or multi-pass optimization. Structurally, the paper lacks smooth transitions between sections, resulting in a fragmented flow from methodology to results. Formatting inconsistencies, grammatical errors, and overly complex phrasing further hinder readability. The absence of statistical validation and limited quantitative rigor reduce the scientific reliability expected in a materials engineering study.

Although this research addresses a relevant problem and presents interesting experimental observations, the paper lacks stronger justification of experimental design, deeper quantitative and theoretical analysis, clearer differentiation from existing literature, and a more concise and cohesive presentation.

About references, many sources are outdated and mostly from regional journals, with very few recent or international studies included. This makes the research appear narrow and not up to date (more on 2025) with the latest advances in friction stir welding, such as thermal modelling, hybrid techniques, or AI-based optimization. The citation style is inconsistent, with errors in formatting, punctuation, and even incorrect publication years. Some references are not properly integrated into the discussion, being mentioned only once without explaining how they support the findings. Important areas like temperature distribution, residual stress, and microstructure modelling are also missing from the cited works. The references need to be updated with more current, high-quality international studies, consistently formatted, and clearly connected to the paper's analysis.
