# Supplementary material for: Influence of pin length on interfacial characteristics and tensile properties of friction-stir-butt-welded AA1060/Q235 clad plates
Source: PLoS One. 2026 Apr 8;21(4):e0346802. doi: 10.1371/journal.pone.0346802 (PMC13061233; doi:10.1371/journal.pone.0346802)
Supplement: Attachment — Submitted filename: Response to Reviewers.doc [file pone.0346802.s002.doc]

To: The Editorial Office of PLOS ONE

Re: Manuscript ID: PONE-D-25-50482–Effect of Tool Pin Length on Microstructure and Mechanical Properties of Friction-Stir-Butt-Welded AA1060/Q235 Steel Clad Plates

Dear Editor and Reviewers,

We sincerely thank the Academic Editor and the Reviewers for dedicating your valuable time and effort and for providing highly constructive and insightful comments. These suggestions have significantly helped us improve the rigor, clarity, and scholarly value of our work. We have carefully and thoroughly revised the manuscript according to every point raised. This letter aims to provide a point-by-point response to all comments and detail the specific modifications made.

**Part 1:** Responses to the Academic Editor’s Journal Requirements

**Editor’s Comment 1:** Please ensure that your manuscript meets PLOS ONE's style requirements.

Response and Action: We have carefully reviewed and adhered to the PLOS ONE style templates, thoroughly checking and adjusting the overall formatting, file naming, etc., of the manuscript to ensure full compliance with the journal's requirements.

**Editor’s Comment 2:** Funding information should not appear in the Acknowledgments; please remove and update the Funding Statement online.

Response and Action: We have completely removed the funding information (“This work was also supported by the Middle-aged and Young Teachers' Basic Ability Promotion Project of Guangxi (2023KY0824).”) from the Acknowledgments section in the manuscript (see Lines 316-317 on page 21). We hereby request to update the Funding Statement in the online submission system to: “This work was supported by the Middle-aged and Young Teachers' Basic Ability Promotion Project of Guangxi (2023KY0824). URL: http://jyt.gxzf.gov.cn/. The funders had no role in study design, data collection and analysis, decision to publish, or preparation of the manuscript.”

**Editor’s Comment 3:** Figures 2, 3, 5, 6, and 10 may contain copyrighted images; permission under CC BY 4.0 is required or they must be replaced.

Response and Action: We confirm that all figures presented in this manuscript (Figs. 1–12) are original and were created by the authors. This includes the tool geometry diagram (Fig. 2), outside view of weld (Fig. 3), SEM macrographs (Fig. 4), interfacial microstructure (Fig. 6), SEM micrographs and EDS line scan results (Fig. 7)， XRD patterns (Fig. 8), fractographs (Fig. 12) and associated data plots (Figs.1, 5, 9, 10, 11). No third-party copyrighted material has been used. Consequently, all figures are eligible for publication under the CC BY 4.0 license, and a statement to this effect has been added to each figure caption.

Editor’s Comment 4: Regarding citations suggested by reviewers, there is no requirement to cite them unless indicated by the editor.

Response and Action: We have critically evaluated the relevant literature suggested by the reviewers. In the revised manuscript, we have cited recent, high-quality publications that are directly relevant and significantly enhance the discussion (e.g., References [1]-[3], [10]-[13]). Those not cited were deemed less central to the core novelty of our study.

**Part 2: Responses to Reviewer #1**

General Comment: Reviewer #1 considered the topic important but pointed out several areas requiring improvement to enhance scientific rigor and impact.

**Comment 1.1:** The abstract, while detailed, is overly descriptive and includes numerical results that should instead appear in the main text. It also does not sufficiently highlight the novelty or distinct contribution of this work relative to existing studies.

Response and Action: We are deeply grateful for all specific comments and have endeavored to address them comprehensively. The Abstract has been completely rewritten (as shown in Lines 9-20 on Page 2 of the revised manuscript).

Action 1: Removed specific numerical results (e.g., “0.38μm”), replacing them with more mechanistic descriptions emphasizing the “critical role” and “identified optimal parameter range.”

Action 2: Explicitly stated the novelty upfront: “applies friction stir welding (FSW) to achieve butt joints of Al/steel clad plates for the first time.”

Action 3: Strengthened the significance, stating the study “reveals the interfacial bonding mechanism… providing theoretical foundations and experimental guidance.”

**Comment 1.2:** The introduction provides an extensive background on Al/steel composites but dedicates excessive space to basic information rather than clearly identifying the research gap. Furthermore, the claim that studies on Al/steel clad plate friction stir welding remain preliminary is not convincingly supported with up-to-date literature. The literature review, embedded within the introduction, lacks critical comparison and fails to synthesize previous findings into a coherent research context. It does not adequately clarify what remains unknown about the role of pin length or how this work advances beyond prior studies. Emerging developments such as hybrid, refill, and ultrasonic-assisted friction stir welding are also not discussed, resulting in a narrow technological scope.

Response and Action: We thank the reviewer for these insightful points. The Introduction has been substantially rewritten and reorganized （as shown in Lines 22-76 on Pages 2-5）.

Action 1: Streamlined the basic background on clad plate applications, focusing the opening on engineering demands and the limitations of conventional welding (see Lines 23-33 on Pages 2-3).

Action 2: By reviewing a large number of domestic and international literatures, it is found that there are few studies focusing on the welding of butt joints in aluminum/steel clad plates. However, the abundant experience gained from dissimilar aluminum-steel FSW can be used for reference, thereby more clearly defining the research gaps and innovations of this study (see Lines 34-39 on Page 3 ).

Action 3: Enhanced the critical literature review. Added new paragraphs systematically reviewing key process parameters (e.g., rotation speed, tilt angle) and derivative technologies (e.g., ultrasonic-assisted, magnetic pulse, arc-enhanced hybrid FSW). We explicitly note the shortcomings in prior research regarding the “systematic influence of tool pin length” and “quantitative correlation between IMC thickness and joint strength” (see Lines 40-68 on Pages 3-4), thereby positioning our study within a broader and more contemporary technological context.

**Comment 1.3:** The experimental section provides clear procedural descriptions but lacks justification for key parameter selections, including rotational speed, traverse rate, and pin geometry. While the three-pass FSW strategy and groove design are described in detail, their optimization rationale and validation are not addressed. The absence of discussion on sample size, error margins, or data reproducibility weakens the experimental credibility. Additionally, important factors such as tool material, heat input estimation, and temperature control are not reported, making it difficult to assess the thermal–mechanical environment influencing IMC formation.

Response and Action: We sincerely thank the reviewer for these suggestions to enhance experimental rigor. Significant additions have been made to the “Experiment” section (see Lines 77-132 on Pages 5-8).

Action 1: Explicitly stated the FSW tool material (H13 tool steel) and treatment (surface quenching) in the text and provided an illustration of the tool geometry (see Lines 106-107 and Fig 2. ).

Action 2: Explained that the main FSW parameters were initially selected based on relevant literature [14-16] and finalized through optimization via preliminary experiments (testing three different rotation speeds and three welding speeds) (see Lines 113-115 on Page 7).

Action 3: Added the heat input estimation formula Q=ηkω^2^/v and explained that the three-pass strategy was adopted to ensure reliable interfacial bonding, based on the observation of lack-of-fusion defects in preliminary one-pass and two-pass welds (see Lines 108-111 on Page 7). The design of the weld groove dimensions was based on temperature numerical simulation, aiming to restrict Al diffusion and the consequent formation of IMC (see Lines 83-86 on Page 5).

Action 4: Added a statement on data reliability: “To evaluate the reliability of the results, three specimens were fabricated and tested under each set of process parameters, with the final results presented as the average value (see Lines 121-122 on Page 8).”

**Comment 1.4:** In the results and discussion section, the authors present valuable microscopic, EDS, and XRD analyses; however, the interpretation remains largely qualitative. Relationships among IMC thickness, pin depth, and tensile strength are mentioned but not statistically analyzed or modelled. Figures are informative but often lack proper labeling, scale bars, or consistent referencing. The mechanical testing results are limited to tensile strength without detailed stress–strain analysis, error bars, or modeling support. Fracture analysis relies solely on visual SEM inspection, with no complementary quantitative data such as elongation, toughness, or energy absorption to substantiate claims. The discussion also lacks integration of findings into broader theoretical or industrial frameworks, leaving the applicability of the results unclear.

Response and Action: We fully agree on the need for more quantitative analysis and deeper discussion. Several additions and modifications have been made.

Action 1: We added an analysis of “stress-strain curves” (new Fig 11 and corresponding discussion), exploring the elastic modulus and plastic deformation behavior under different pin lengths, providing deeper mechanical insight (see Lines 272-279 on Pages 18-19).

Action 2: When discussing tensile strength variations, we explicitly added the corresponding elongation data for each specimen (e.g., 33.1% elongation at 3.8 mm pin length), directly linking fracture morphology (ductile/brittle) with quantitative mechanical properties (see Lines 280-292 on Page 19).

Action 3: To visualize the relationship between key variables more intuitively, we integrated the data to add a new “Figure 9: Variations in tensile strength, ETs, and interfacial IMC thickness as a function of tool pin length.” This provides a graphical representation of the multi-variable interplay, strengthening the quantitative basis of the discussion (see Lines 225-244 on Pages 15-16).

Action 4: In the “Conclusion” section, we strengthened the practical implications, offering clear engineering guidance: “it is recommended to control the FSW pin length to just contact the Al/steel interface.” We also acknowledged the study’s limitation (restricted parameter range) and suggested future work (expanding the process window for more comprehensive models), thus connecting our findings to wider practical and theoretical frameworks (see Lines 311-314 on Page 21).

Action 5: We have standardized the style of the scale bars in the figures (e.g., Figures 4, 6, 7, and 12) and made consistent the references to these figures in the text.

**Comment 1.5:** The conclusions mainly restate observations instead of deriving actionable engineering insights or guidelines. There is no discussion of study limitations, measurement uncertainties, or recommendations for future research, such as fatigue behavior, temperature field simulation, or multi-pass optimization. The overall structure would benefit from smoother transitions between sections to improve narrative flow. Numerous minor grammatical issues, inconsistent terminology, and formatting errors further affect readability.

Response and Action: Following the modifications related to Comment 1.4, the Conclusion has been rewritten (see Lines 297-314 on Pages 20-21). The new conclusion first states the core finding that “tool pin length is a critical parameter,” then elaborates on the “performance under optimal parameters” and “failure mechanisms under insufficient/excessive parameters,” (see Lines 298-310 Pages 20-21) and finally provides clear engineering recommendations, study limitations, and future work directions, making it more insightful and guiding.Practical guidance was added: position the pin tip at the Al/steel interface to optimize IMC thickness and steel-side integrity.A limitation is the narrow process window examined. Future studies should expand the parameter range to develop more general models (see Lines 311-314 on Page 21).

Comment 1.6: Regarding references, the paper relies heavily on older or regionally focused sources, with few recent international studies cited. This gives the impression that the research is not up to date with current advances in friction stir welding, such as hybrid methods, thermal–mechanical modeling, or AI-based parameter optimization. Several references are cited without sufficient explanation of their relevance, and inconsistencies in citation formatting and publication details are evident. The reference list requires significant revision to include more recent, high-quality works and ensure consistency in style.

Response and Action: We have comprehensively updated the reference list.

Action 1: Removed some older or regionally focused references and added multiple recent, high-quality international journal articles, particularly on advanced topics like dissimilar material FSW, ultrasonic-assisted FSW, and magnetic pulse welding (e.g., new References [1-3], [6] [10-12]，[14-16]).

Action 2: Unified and rigorously checked the formatting of all references to comply with journal style.

**Part 3: Responses to Reviewer #2**

**Comment 2.1:** On what basis were the process parameters in Table 2 chosen?

Response and Action: The parameters (TIG/SMAW) were selected based on the standard specifications for the welding consumables (ER50-6, E4303), the plate thickness (8 mm steel substrate), and conventional welding practice to achieve defect-free fusion welds. TIG was used for the root pass to ensure root quality, and SMAW was used for filling and capping passes to improve efficiency. The interpass temperature was controlled below 200°C to prevent overheating of the aluminum side of the clad plate. A concise explanation has been retained in the text (see Lines 99-102 on Page 6).

**Comment 2.2:** Basis on which pin diameter in Table 3 were chosen?

Response and Action: The tool pin geometry (including diameter) was determined based on extensive prior literature（[14-16]） on FSW of dissimilar Al/steel materials. A relatively small pin diameter helps achieve sufficient material stirring without excessively increasing the thermomechanical input to the steel region, thereby controlling IMC growth. We have added an illustration of the tool geometry (Figure 2) and mentioned that parameter selection was informed by relevant literature in the “Experiment” section (see Line 114 on Page 7).

**Comment 2.3:** Micrograph showing the dynamically recrystallized region may be added.

Response and Action: This is an excellent suggestion. In Figures 6(f) of the revised manuscript, we have indicated regions near the steel region interface exhibiting “incomplete DRX, incomplete DRX, fibrous texture.” The grains in DRX are finer and more equiaxed compared to the base metal, evidencing DRX due to thermomechanical effects. We have explicitly noted this feature in the figure caption and textual description (see Lines 174-176 on Page 11).

**Comment 2.4:** Mechanical properties of the base materials need to be added.

Response and Action: We fully agree. We have added a new Table 2: Mechanical properties of Al/steel clad plates and the welding materials in the “Experiment” section, listing the tensile strength, yield strength, and elongation for the AA1060/Q235 steel clad plate, and the welding consumables used (ER50-6, E4303) (see Line 91 on Pages 5-6).

**Concluding Remarks**

Once again, we express our deepest gratitude to the Editor and the Reviewers for your diligent work and invaluable guidance. Your comments have made this study more robust, clear, and complete. We hope the revised manuscript now satisfactorily addresses all the points raised and look forward to your further evaluation.

Sincerely,

The Authors:

Dengfeng Jia, Wei Li*, Tenghui Xia

Guilin University of Aerospace Technology

2025.12.25
