# Supplementary material for: Influence of pin length on interfacial characteristics and tensile properties of friction-stir-butt-welded AA1060/Q235 clad plates
Source: PLoS One. 2026 Apr 8;21(4):e0346802. doi: 10.1371/journal.pone.0346802 (PMC13061233; doi:10.1371/journal.pone.0346802)
Supplement: Attachment — Submitted filename: Response to Reviewers.docx [file pone.0346802.s003.docx]

**Manuscript ID:** PONE-D-25-50482R1

**Title:** Influence of Pin Length on Interfacial Characteristics and Tensile Properties of Friction-Stir-Butt-Welded AA1060/Q235 Clad Plates

**Journal:** PLOS ONE

Dear Dr. Sharma and Reviewers,

We sincerely thank you for your thorough and constructive review. Your insightful comments have been invaluable in improving the quality and rigor of our work. We have carefully considered all the points raised and revised the manuscript accordingly. Below, we provide a point-by-point response to each comment, detailing the changes made in the revised manuscript .

## Response to Reviewer #1

### **General Comment**

The reviewer provides a comprehensive assessment, noting that the manuscript addresses a relevant topic and presents clear experimental trends, but is constrained by qualitative interpretation, limited statistical rigor, and overextended mechanistic claims. The reviewer suggests that the study is suitable as an exploratory investigation but requires deeper quantitative analysis and stronger validation.

****Our response:**** We gratefully acknowledge the reviewer's balanced and constructive assessment. We have taken all comments seriously and substantially revised the manuscript to address each point. The revisions focus on: (1) tempering overstated novelty and mechanistic claims, (2) explicitly acknowledging experimental limitations, (3) strengthening statistical presentation, (4) providing more critical comparison with existing literature, and (5) reframing conclusions as correlations rather than validated mechanisms. Detailed responses follow below.

### Comment 1.1: Title and Scope

**Reviewer comment:** "The title and overall scope of the manuscript somewhat overstate the generality and novelty of the work. While the title highlights the 'effect of tool pin length,' the experimental design inherently couples pin length with pin penetration into the steel substrate, making it difficult to isolate pin length as a truly independent parameter. Furthermore, claims such as 'for the first time' are not sufficiently substantiated through a systematic comparison with existing studies on Al/steel butt or clad plate welding. Given the narrow parameter window, single material system, and fixed tool geometry, the conclusions are not readily generalizable, yet the title implies broad applicability."

****Our response:**** We thank the reviewer for this important observation. We fully agree that the original manuscript overstated novelty and generality. We have made the following revisions:

1.1.1 ****Title revision****

We have changed the title to be more specific and to reflect the exploratory nature of the work:

**Original:** "Effect of Tool Pin Length on Microstructure and Mechanical Properties of Friction-Stir-Butt-Welded AA1060/Q235 Steel Clad Plates"

**Revised:** "Influence of Pin Length on Interfacial Characteristics and Tensile Properties of Friction-Stir-Butt-Welded AA1060/Q235 Clad Plates"

The revised title focuses on "interfacial characteristics" and "tensile properties" rather than the broader "microstructure and mechanical properties," thereby avoiding any implication of broad applicability.

****1.1.2 Removal of "for the first time"****

We have removed the instance of "for the first time" from the abstract (Abstract, Lines 30-31).

****1.1.3 Explicit acknowledgment of variable coupling****

In the introduction, we now clearly state:

"In our experimental setup, pin length and its penetration depth into the steel are coupled, so the 'influence of pin length ' discussed here inherently reflects this combined effect." (Introduction, Lines 83-84)

****1.1.4 Acknowledgment of limited generalizability****

In the conclusion, we explicitly note:

"It is important to note that in this study, pin length and penetration depth are inherently coupled. Therefore, the observed optimal pin length is specific to the fixed parameters (e.g., plunge depth, tool geometry) used here and may not directly transfer to other processing conditions or material systems." (Conclusion, Lines 316-319)

### Comment 1.2: Abstract

**Reviewer comment:** "The abstract, although improved, still places excessive emphasis on mechanistic claims and optimality without adequate contextualization of experimental limitations. Assertions regarding the revelation of 'interfacial bonding mechanisms' are not matched by direct mechanistic validation through modeling, in-situ measurements, or kinetic analysis. The mention of an 'optimal parameter range' remains vague, as no tolerance, robustness, or sensitivity to process variations is discussed. Additionally, key limitations, such as the restricted pin length range and the absence of service-relevant mechanical testing, are not acknowledged."

****Our response:**** We agree that the abstract overreached in its mechanistic claims. We have substantially revised the abstract to be more circumspect and to acknowledge limitations:

****1.2.1 Revised abstract****

"This study explores friction stir butt welding of AA1060/Q235 clad plates to address the chemical industry's need for robust aluminum/steel clad joints. To manage the formation of intermetallic compounds, we employed FSW tools with varying pin lengths, enabling simultaneous welding of the interfacial transition zone and the cladding fusion zone. The pin length significantly influenced joint tensile strength by affecting three key factors: the thickness of the IMC layer, the effective thickness of the steel region, and interfacial microcracking. Optimal tensile strength was achieved when the pin length was adjusted so that its tip precisely reached the Al/steel interface. Although the study is constrained by the number of process parameters, the observed trends illuminate the initial correlations between processing conditions, interfacial characteristics, and mechanical performance. This underscores the necessity of balancing IMC growth with interfacial integrity." (Abstract, Lines 29-39)

**Key changes:**

Removed "for the first time" and "reveals the interfacial bonding mechanism"

In response to the reviewer's concern about the vagueness of "optimal parameter range," we have revised the abstract to state a specific optimal condition: "Optimal tensile strength was achieved when the pin length was adjusted so that its tip precisely reached the Al/steel interface." (Abstract, Lines 35-36)

Added explicit acknowledgment of limitations: "Although the study is constrained by the number of process parameters". (Abstract, Lines 36-37)

Used "initial correlations" rather than "mechanisms" to reflect the exploratory nature. (Abstract, Lines 37-39)

****1.2.2 Acknowledgment of the need for service-relevant testing:****

In the discussion, we now note:

"While this study focused on tensile behavior, a comprehensive understanding of joint performance would require additional testing, including evaluations of shear strength and fatigue life." (Results and discussion, final paragraph of fracture section, Lines 295-297)

### Comment 1.3: Introduction and Research Gap

**Reviewer comment:** "The introduction provides extensive background information but does not sufficiently sharpen the research gap. The novelty is framed largely by the scarcity of prior work rather than by a clearly defined unanswered scientific or engineering question. Although derivative FSW techniques such as ultrasonic- and arc-assisted methods are mentioned, they are not critically compared to conventional FSW in terms of scalability, cost, or industrial feasibility. The literature review remains largely descriptive and lacks synthesis, with limited discussion of conflicting results or theoretical frameworks related to IMC growth, heat generation, and material flow in Al--steel systems."

****Our response:**** We thank the reviewer for this insightful critique. We have substantially revised the introduction to address each of these concerns, as detailed below.

****1.3.1 Sharpened the research gap****

Rather than merely noting the scarcity of prior work, we now frame the research gap as a clearly defined unanswered scientific question grounded in engineering relevance. The introduction now explicitly states:

**First:** The practical challenge of achieving reliable Al/steel clad plate joints underscores the engineering significance of this work. "Achieving adequate tensile strength and robust interfacial bonding in butt-welded Al/steel clad plates for chemical vessels remains challenging." (Introduction, Lines 65-66)

**Second:** Within this context, the complete absence of FSW studies on Al/steel clad plate butt joints (the ITZ and CFZ have never been welded simultaneously) and the unexplored influence of pin geometry for clad plate configurations define the specific scientific knowledge gap. "Despite these insights, simultaneous friction stir welding of the interfacial transition zone (ITZ) and cladding fusion zone (CFZ) in Al/steel clad plate butt joints has not been reported, let alone the influence of pin geometry on interfacial microstructure, IMC thickness, and mechanical properties." (Introduction, Lines 78-81)

This dual framing transforms the novelty from a passive observation ("studies are scarce") into an active, engineering-relevant scientific question that our study directly addresses.

**1.3.2 Critical comparison of derivative techniques**

We have restructured the paragraph on derivative FSW techniques to move beyond mere description and provide critical evaluation. The revised paragraph now:

"Recent advances in FSW derivatives—arc‑enhanced hybrid, magnetic field–assisted, and ultrasonic‑assisted processes—have improved material flow and suppressed brittle phases. Song [10] reported that ultrasonic‑assisted FSW produced a continuous ~1.2 μm IMC layer in Al–steel lap joints and markedly increased joint strength, although the method raised equipment and process complexity. Liu et al. [11] showed that ultrasonic assistance enhances lap‑joint formability and performance but did not examine how tool geometry interacts with ultrasonic energy. Li et al. [12] achieved high‑quality welds between aluminum alloy and stainless steel tubes using magnetic pulse welding; that technique, however, relies on an axisymmetric electromagnetic field and is not directly applicable to plate butt joints. Liu et al. [13] applied arc‑enhanced hybrid FSW to Al/steel lap joints and observed that joint strength rose and then fell as pin length increased, but they did not quantify the relationship between IMC thickness and strength. Overall, compared with conventional FSW these approaches entail higher equipment costs, greater process complexity, and incompletely understood field‑coupling mechanisms. These limitations motivate a focused investigation of conventional FSW parameters—such as pin length—in Al/steel clad plate butt joints. " (Introduction, Lines 51-64)

**Key improvements:**

Each derivative technique is presented with both its potential and its limitations.

The limitations explicitly address scalability, cost, and feasibility (e.g., "raised equipment and process complexity," "relies on an axisymmetric electromagnetic field," "not directly applicable")

The paragraph concludes by synthesizing these limitations into a coherent motivation for studying conventional FSW parameters

**1.3.3 Synthesis of literature**

Rather than simply describing individual studies, we now synthesize findings to establish general principles and identify knowledge gaps:

"Ma et al. [7] reviewed the optimization of FSW parameters to obtain an appropriately thick IMC layer and noted that this promotes Al/steel joining. Kaushik et al. [8] reported that larger tool shoulder and pin diameters increase IMC thickness because they generate higher frictional heat. Chitturi et al. [9] demonstrated that tool tilt angle and pin penetration depth influence IMC layer thickness and distribution, thereby affecting joint strength and fracture mode. Together, these studies show that welding parameters and tool geometry control joint quality by regulating heat input and, consequently, IMC layer thickness at the interface. Excessive heat input thickens the IMC layer, while insufficient heat leads to poor bonding or lack of fusion; both outcomes markedly reduce tensile strength. However, this understanding is based mainly on studies of Al–steel butt or lap joints in monolithic plates, and investigations addressing FSW of Al/steel clad plate butt joints are still scarce. " (Introduction, Lines 67-78)

This structure achieves:

**Synthesis:** The three studies are not just listed but integrated into a coherent principle ("Together, these studies show...").

**Generalization:** A universal relationship (heat input → IMC thickness → joint quality) is established

**Gap identification:** The "However" clause clearly delineates what is known (monolithic plates) versus what remains unknown (clad plates)

### Comment 1.4: Experimental Design and Methodology

**Reviewer comment:** "From a methodological standpoint, the experimental design lacks sufficient justification and transparency. While the authors state that welding parameters were optimized through preliminary trials, no supporting data or selection criteria are provided. Tool geometry parameters other than pin length, such as pin diameter and shoulder design, are fixed without justification, despite their known influence on heat input and material flow. Heat input estimation relies on a simplified empirical formula, yet no experimental temperature measurements or validation are presented. The use of a three-pass FSW strategy introduces cumulative thermal effects, but inter-pass microstructural evolution is not examined. Although three specimens per condition were tested, no statistical analysis, error bars, or confidence intervals are reported, weakening claims of repeatability and optimality."

****Our response:**** We thank the reviewer for identifying these methodological gaps. We have substantially expanded the experimental section to address each point.

****1.4.1 Justification for parameter selection****

First, we now clarify that the fixed parameters (tool tilt and plunge depth) were chosen based on established practice in the literature:

"Consistent with previous studies [14–16], we maintained a fixed tool tilt of 2.5° and a plunge depth of 0.2 mm to ensure proper shoulder contact and forging, which are typical in Al-alloy FSW." (Experiment, Lines 121-123)

Second, we now describe the preliminary optimization trials and the criteria used to select the final welding speed and rotational rate:

"We varied the rotational speed (1000, 1200, 1400 rpm) and welding speed (120, 150, 180 mm/min) to optimize surface quality. Based on the observation of a consistent, defect-free surface at the cladding fusion zone, we selected 1200 rpm and 150 mm/min for subsequent welding." (Experiment, Lines 123-125)

While we acknowledge that detailed data from these preliminary trials are not included, the selection criterion—a consistent, defect-free surface—is clearly stated, providing transparency regarding how the final parameters were chosen.

**1.4.2 Justification for fixed tool geometry**

The reviewer correctly notes that tool geometry parameters other than pin length (shoulder diameter, pin diameter, pin shape) were fixed without explicit justification. We have now added this justification:

To avoid confounding effects on heat input and material flow, we fixed the shoulder diameter, pin diameter, and pin shape (tapered threaded). (Experiment, Lines 111-113)

This statement explains that these parameters were held constant specifically to isolate the effect of pin length as the primary variable of interest. By fixing these parameters, we ensure that any observed changes in joint characteristics can be attributed to pin length rather than to uncontrolled variations in tool geometry.

**1.4.3 Acknowledgment of limitations in heat input estimation**

We have clarified in the experimental section that the FSW heat input was estimated using the empirical formula Q=ηKω2/υ, while acknowledging that actual interface temperatures may vary due to differences in thermal conductivity and contact conditions at the Al/steel interface (Experiment, Lines 113-115). As stated in the experimental section: "Simulations of the temperature field during butt welding indicated that, on the steel-side first pass, temperatures dropped below 300 °C beyond 5 mm from the weld centerline" (Experiment, Lines 90-91). Although direct temperature measurements were not performed, these numerical simulations provided valuable guidance for groove design. Recognizing the need for experimental validation, we have added a statement in the discussion: "The relationship between heat generation and pin length warrants validation through direct temperature measurements in future work" (Macrostructure of Weld Appearance, Lines 163-164).

**1.4.4 Three-pass FSW strategy**

The reviewer raises a valid concern about cumulative thermal effects from the three-pass strategy and the lack of examination of inter-pass microstructural evolution. We have added justification for the three-pass approach:

Since both one-pass and two-pass trials consistently failed to achieve fusion, we implemented three passes to ensure robust Al/steel bonding [Fig. 1(d)]. (Experiment, Lines 116-117)

This explains that the three-pass strategy was not arbitrary but was necessitated by the failure of fewer passes to achieve adequate bonding. We acknowledge that this introduces cumulative thermal effects, and we have added this point to the limitations section in the microstructure of joint interface:

While the preceding discussion has addressed the interfacial features governing mechanical performance, the cumulative thermal effects of the three-pass strategy and their microstructural consequences between passes have yet to be investigated. Future work should examine this inter-pass evolution to clarify its role in determining final joint properties.(Microstructure of Joint Interface, Lines 220-224)

**1.4.5 Statistical analysis**

The reviewer correctly notes that although three specimens per condition were tested, no statistical analysis, error bars, or confidence intervals were reported. We have comprehensively addressed this throughout the manuscript.

First, in the experimental section, we now explicitly state:

"For each parameter set, three specimens were tested, with tensile strengths reported as mean ± SD." (Experiment, Line 128)

Second, throughout the Results and discussion section, all tensile strength values are now presented with their standard deviations:

"At a pin length of 3.6 mm, the strength reached 367.7 ± 0.37 MPa." (Mechanical properties, Lines 227-228)

"The peak strength of 384.3 ± 0.57 MPa was observed at 3.8 mm." (Mechanical properties, Lines 231-232)

"Beyond this optimal length, tensile strength progressively declined as pin length increased from 4.0 mm to 4.4 mm (365.3 ± 0.12 MPa to 361.2 ± 0.30 MPa)." (Mechanical properties, Lines 237-238)

Third, the relevant Fig. 9 now include error bars representing standard deviations, providing visual representation of data variability.

These additions provide the statistical transparency that was previously missing and strengthen the claims of repeatability and optimality.

### Comment 1.5: Macrostructural Analysis

**Reviewer comment:** "The macrostructural analysis of weld appearance is superficial. Surface morphology is declared insensitive to pin length based solely on visual inspection, without quantitative evaluation of flash formation, surface roughness, or defect density. This limits the ability to correlate surface features with subsurface material flow and thermal conditions."

****Our response:**** We agree that the macrostructural analysis was qualitative. We have revised this section to acknowledge this limitation and propose future work:

In the "Macrostructure of weld appearance" section, we have added the following sentence:

"Further work is needed to quantify surface formation more precisely."(Macrostructure of weld appearance, Lines 139-140)

**Explanation:** The primary objective of this section was to assess whether pin length visibly affected weld surface characteristics, which we found not to be the case. We fully agree with the reviewer that quantitative evaluation of flash formation, surface roughness, and defect density would strengthen the analysis and enable correlation with subsurface phenomena. The added sentence explicitly recognizes this need and positions it as a priority for future investigations.

### Comment 1.6: Microstructural Analysis

**Reviewer comment:** "Similarly, the microstructural analysis is dominated by qualitative observations. Claims regarding grain refinement, dynamic recrystallization, and fibrous textures are not supported by quantitative techniques such as EBSD. The identification of intermetallic compounds relies on limited EDS point analyses and a single XRD pattern, which is insufficient to confirm phase continuity or spatial variability along the weld."

****Our response:**** We thank the reviewer for this insightful and constructive critique. We fully acknowledge the limitations of our microstructural analysis and have taken several steps to address these concerns in the revised manuscript.

**1.6.1 Acknowledgment of EBSD absence**

We acknowledge that EBSD analysis would provide valuable quantitative data on grain orientation, recrystallization fractions, and texture evolution—insights that could significantly deepen the understanding of microstructural evolution in these joints. While such analysis was beyond the scope of the present study, we recognize its importance and have identified it as a priority for future investigations in the Microstructure of Joint Interface section.

A magnified view of the steel-side microstructure [Fig. 6(f)] reveals fibrous texture, DRX zones, and regions of incomplete DRX. These microstructural features warrant quantitative EBSD characterization of grain orientation, recrystallization fractions, and texture evolution. (Microstructure of Joint Interface, Lines 182-184)

**1.6.2 EDS and XRD limitations**

The reviewer correctly notes that EDS point analyses sample only discrete locations and that a single XRD pattern does not reveal phase continuity or spatial variability. We have explicitly acknowledged this limitation in the manuscript by adding the following sentence immediately after presenting the XRD results:

However, EDS point analyses sample only discrete locations, and a single XRD pattern confirms the presence of these phases but does not reveal their spatial distribution or continuity along the entire weld interface.(Microstructure of Joint Interface, Lines 197-199)

### Comment 1.7: IMC Formation and Microcrack Development

**Reviewer comment:** "The discussion of IMC formation and microcrack development, while plausible, remains largely interpretative. Microcracks are attributed to thermal stress and IMC brittleness without residual stress measurement, thermal gradient analysis, or fracture mechanics considerations. The relationship between pin length, heat input, IMC thickness, and joint strength is presented as correlative rather than causal, as no regression analysis or mechanistic modeling is provided. Consequently, the proposed mechanisms remain hypotheses rather than validated explanations."

**Our response:** We thank the reviewer for this important observation. We have revised the discussion to frame our interpretations more modestly as hypotheses rather than validated mechanisms, and we have explicitly called for future work to provide the necessary validation.

**1.7.1 Cautious language**

**We have carefully reviewed the discussion and replaced definitive causal statements with language that appropriately reflects the interpretative nature of our observations. Specific revisions include:**

**This increase likely results from greater frictional heat at deeper penetrations, which promotes more extensive Al-Fe interdiffusion and the growth of a brittle IMC layer. Thermal stresses during cooling may then induce cracking within this layer. (Microstructure of Joint Interface, Lines 187-190)**

**This broadening is primarily attributed to the thickening of brittle IMC layers and a higher proportion of incompletely dynamically recrystallized grains.** (Mechanical properties, microhardness section, Lines 267-269)

Microcracks appear in the steel region adjacent to the interface, as shown in Figs. 7(c1) and (d1), likely resulting from mismatched thermomechanical conditions between frictional heating and mechanical stirring. (Microstructure of Joint Interface, Lines 217-219)

****1.7.2 Explicit acknowledgment of correlative nature****

**We agree with the reviewer that the relationships observed in this study are correlative rather than causal. In the mechanical properties section, we have added a sentence that explicitly states this limitation:**

**Although these findings suggest a correlation among pin length, interfacial microstructure, and tensile strength, further investigation is needed to establish a clearer causal model. (Mechanical properties, Lines 242-243)**

****1.7.3 Future work for validation****

**The reviewer correctly notes that validating the proposed mechanisms would require additional measurements and analysis. We have incorporated this recognition into the manuscript by adding specific suggestions for future investigation:**

**This will include residual stress measurements and a detailed examination of crack propagation mechanisms within the IMC layer. (Mechanical properties, Lines 243-245)**

### Comment 1.8: Mechanical Property Evaluation

**Reviewer comment:** "The mechanical property evaluation focuses almost exclusively on uniaxial tensile strength. While stress--strain curves and elongation data are included, key performance metrics relevant to clad plate applications, such as shear strength, fatigue resistance, fracture toughness, and thermal cycling behaviour, are still absent. The fracture analysis is limited to qualitative SEM observations, without quantitative assessment of dimple size, cleavage fraction, or crack initiation sites relative to IMC morphology. This restricts the depth of insight into failure mechanisms."

****Our response:**** We agree that a comprehensive mechanical evaluation would require additional testing. We have revised the manuscript to acknowledge this limitation and to propose future work.

****1.8.1 Acknowledgment of limited mechanical testing****

**We agree that shear strength, fatigue resistance, and other service-relevant properties are essential for a complete understanding of joint performance. While such tests were beyond the scope of the present study—which focused on establishing fundamental correlations between pin length and tensile behavior—we have explicitly acknowledged this limitation in the manuscript. In the final paragraph of the fracture section, we now state:**

**While this study focused on tensile behavior, a comprehensive understanding of joint performance would require additional testing, including evaluations of shear strength and fatigue life.(Mechanical properties, Lines 295-297)**

**The title of the paper has also been changed to: Influence of Pin Length on Interfacial Characteristics and Tensile Properties of Friction-Stir-Butt-Welded AA1060/Q235 Clad Plates**

****1.8.2 Quantitative fracture analysis****

**We agree with the reviewer that quantitative fracture analysis would provide deeper insight into failure mechanisms.** While we cannot retrospectively quantify dimple size or cleavage fraction, we have made our fracture descriptions more quantitative where possible:

At 4.0 mm [Fig. 12(b)], the fracture mode transitioned to a mixed ductile–brittle type, featuring cleavage river patterns and shallow dimples, with cleavage facets covering about 30% of the surface. (Mechanical properties, Lines 283-285)

In both cases, cleavage facets make up over 98% of the fracture surface. The elongation values are 24.7% and 22.3%, respectively, which are indicative of brittle fracture. (Mechanical properties, Lines 293-294)

### Comment 1.9: Discussion, Conclusions

**Reviewer comment:** "The discussion and conclusions largely restate experimental observations rather than synthesizing broader engineering principles. Although a recommended pin length is proposed, no tolerance range or robustness assessment is provided, limiting practical applicability. While limitations are briefly acknowledged, they are not critically explored, and the implications of these limitations for industrial deployment remain unclear. Despite improvements to the reference list, the manuscript still underrepresents computational, modeling, and mechanism-driven studies, and several citations are used to support general statements rather than specific arguments."

****Our response:**** We thank the reviewer for this thoughtful and constructive critique. We agree that the original discussion and conclusions were overly descriptive and lacked synthesis. To address these concerns, we have substantially revised the manuscript, with specific changes detailed below.

****1.9.1 Synthesis into broader principles****

**Rather than merely restating experimental observations, the conclusions now synthesize the findings into three overarching principles that capture the essential relationships governing joint performance:**

**(1) Joint strength is determined by the interplay between interfacial metallurgical bonding and the load-bearing capacity of the steel side. Pin length plays a crucial role in this balance through two coupled mechanisms: the pin's entry into the steel substrate determines the steel's effective thickness; and deeper penetration with longer pins enhances heat input and mechanical stirring, influencing the IMC layer thickness, elemental diffusion distance, and microcrack density at the interface.**

**(2) Optimal balance is achieved when the pin tip precisely contacts the Al/steel interface. At this length, a continuous submicron IMC layer forms, the steel maintains its full thickness without microcracks, and the joint attains maximum tensile strength. Fracture on the steel side adjacent to the interface exhibits a ductile nature, characterized by a dimpled morphology.**

**(3) Deviations from this optimal length disrupt the balance. A pin that is too short leaves the steel unplasticized and the interface unbonded; a pin that is too long promotes IMC overgrowth, interfacial microcracking, and reduced steel thickness—all of which lower strength and shift fracture on the steel side to a brittle mode. (Conclusion, Lines 302-314)**

**These principles move beyond simple observation to establish a conceptual framework that connects pin length, interfacial characteristics, and mechanical performance, thereby providing broader engineering insight.**

****1.9.2 Practical guidance with tolerance****

**We agree that practical applicability requires more than a single recommended value. To address this, we have added a tolerance range based on our experimental observations:**

**Therefore, under the fixed processing conditions applied in this study, positioning the pin tip within ±0.1 mm of the Al/steel interface serves as a practical starting point for optimizing the process. (Conclusion, Lines 315-316)**

****1.9.3 Critical exploration of limitations****

**We have expanded the limitations section to critically examine the implications of our findings for broader application:**

**It is important to note that in this study, pin length and penetration depth are inherently coupled. Therefore, the observed optimal pin length is specific to the fixed parameters (e.g., plunge depth, tool geometry) used here and may not directly transfer to other processing conditions or material systems. Future research should focus on decoupling these variables to develop more generalizable process–structure–property models. (Conclusion, Lines 316-321)**

We believe the revisions have substantially improved the manuscript and addressed all of the reviewer's concerns. The English throughout has been carefully revised to meet the journal's standards for clarity and accuracy. The manuscript now presents the work as an exploratory investigation with clear experimental trends, while appropriately acknowledging limitations and framing conclusions as correlations rather than validated mechanisms. We are grateful for the opportunity to revise and hope that the manuscript is now suitable for publication in PLOS ONE.

Thank you for your time and consideration.

Sincerely,

Wei Li
Corresponding Author
Engineering Comprehensive Training Center
Guilin University of Aerospace Technology
Guilin, Guangxi, China
Email: 2024033@guat.edu.cn
